# Interprofessional Education: Reaching Health Professionals With an Interactive Professional Virtual/Online Event on Advocacy and Policy

**DOI:** 10.3389/fpubh.2020.606394

**Published:** 2020-12-01

**Authors:** Karen D. Liller, Zachary Pruitt, Somer Goad Burke

**Affiliations:** College of Public Health, University of South Florida, Tampa, FL, United States

**Keywords:** health professionals' education, interprofessional education, advocacy and policy education, virtual education, working in teams

## Abstract

Competencies in health policy and advocacy should be developed by all health professionals to effectively advance their professions but also effectively collaborate in interprofessional teams to improve public health. However, the COVID-19 epidemic presents a challenge to reaching students of health professions through face-to-face offerings. To meet this need, the University of South Florida College of Public Health developed asynchronous and synchronous online health policy and advocacy modules delivered to an interprofessional group of students pursuing health careers. After learning policy and advocacy material individually through a self-paced online curriculum, faculty gathered the students for a synchronous online event where they formed collaborative groups. In interprofessional teams, students prepared and presented advocacy briefs that were critiqued by the faculty. Post-event evaluation results showed that most students strongly agreed that the interprofessional event was very effective, and they all would recommend the program to other students. Universities and colleges educating students of health professions can take advantage of the technologies employed to keep students safe in the COVID-19 pandemic and still reach students effectively with interprofessional health policy and advocacy content.

## Introduction

Adult learners prefer to be involved in the planning and evaluation of their instruction and to see immediate relevance of learning to their jobs or lives (1). Capturing the attention of adult learners—especially in the online environment—requires a more problem-centered approach to learning, as opposed to learning through passive reception of knowledge (2). Thus, utilization of these principles of adult education can help health professionals to be skilled in advocacy and policy development in an online environment.

However, students of health professions often lack opportunities to build effective policy and advocacy skills due to barriers in health profession education, such as competing priorities, perceived lack of time, weak support from administration, absence of expertise, and logistical impracticalities (3–5). Therefore, approaches to teaching health advocacy and policy in health profession education programs need to be further developed in the literature. There is a growing need for health professions to translate clinical and public health practice skills into design and development of health policy (3, 6). Some published pedagogical literature describing health policy education approaches exists, including descriptions of curriculum (7), case-based learning (8), practicum experiences (9), and semester-long courses (10).

In addition to basic understanding of health policy processes, there is a critical need to build advocacy skills among public health professionals (11). Recognizing that several of our greatest health achievements include clean water, passage of laws limiting toxic substances, laws removing lead from products, and use of seatbelts (12), learning public health advocacy skills is valued as a long-term investment in curricula for many types of health professions (13). Therefore, health profession students need to be taught the importance of successful advocacy of public health issues (14). Hearne (15) provides a sample advocacy course using hands-on learning with didactic and skill building exercises addressing public health areas. Other pedagogical studies reported undergraduate medical curricula that included physician advocacy components focused on population-level injury prevention (16).

## Pedagogical Framework

The Center for the Advancement of Interprofessional Education (CAIPE) defined interprofessional education as “occasions when two or more professions learn together with the object of cultivating collaborative practice” (17). More recently interprofessional education was defined as “when students from two or more professions learn about, from, and with each other to enable effective collaboration and improve health outcomes” (18). The Council on Education for Public Health (CEPH) clarifies the Master of Public Health (MPH) competency with “interprofessional” referring to the “engagement with professionals (either students in other professions or practicing professionals) outside of public health (e.g., architects, nurses), rather than engaging with individuals from other public health disciplines (e.g., biostatisticians, health promotion specialists)” [(19), p.18].

The Institute of Medicine Committee first spoke of the importance of interdisciplinary education in the 1970s. In 2003 the Institute of Medicine Committee on Health Professions Education wrote a report indicating that all health professionals should be educated as members of an interdisciplinary team. The World Health Organization (WHO) (18) issued a call to action for all health and education systems to use interprofessional education and collaborative practice to improve health outcomes. The WHO proposed that through interprofessional education the health workforce would become a collaborative practice-ready workforce and through collaborative practice there would be optimal health services. They analyzed research that found numerous benefits to collaborative practice including improved health outcomes for people with chronic diseases, improved access to care, decreased length of hospital stay and mortality rates, and increased patient satisfaction (18).

The Interprofessional Education Collaborative (IPEC) was formed in 2009 when six associations of schools of health professions joined to encourage and advance interprofessional learning experiences in the preparation and education of health-professional students for team-based care and improvements in population-based health outcomes. The Association of Schools and Programs of Public Health was one of the founding associations. In 2011, the collaborative released the *Core Competencies for Interprofessional Collaborative Practice* which is widely used as the framework for interprofessional education within the health sciences. In 2016 an update was written to organize the competencies (values and ethics, roles and responsibilities, interprofessional communication, and teamwork) into a single domain of Interprofessional Collaboration. The document also includes sub-competencies under each competency (20). In 2016, the Council on Education for Public Health updated their MPH Competencies and included a new competency in support of interprofessional teamwork (19).

Consistent with ASPPH *Core Competencies for Interprofessional Collaborative Practice* (20), the University of South Florida (USF) College of Public Health is a contributing supporter of USF Health's Interprofessional Education and Practice programming, a collaboration that includes the Morsani College of Medicine, College of Nursing, Taneja College of Pharmacy, School of Biomedical Sciences, and School of Physical Therapy and Rehabilitation Sciences. USF Health has offered interprofessional educational programming for over 10 years structured around IPEC's core competencies and each programs' specific competencies. The majority of the interprofessional education programs is offered through in-person modules. However, the need for online opportunities became more pronounced due to the COVID-19 pandemic, resulting in the development of a fully online Health Policy and Advocacy interprofessional education (IPE) module that could be piloted with students from various health professions.

## Learning Environment

For this IPE, three 45-min asynchronous competency-based modules were created on advocacy and policy development. Students completed the asynchronous modules prior to the IPE session. Objectives of the two health policy modules were: (1) understand the relationship of federal and state/local governments in public health law, (2) recognize the dichotomy of common good vs. individual freedoms in public health law, (3) summarize the steps involved in the development of policy, and (4) explain the phases of health policymaking. In the first of the two health policy modules, the fundamentals of public health law are provided to students, including an in-depth discussion of legal powers and duties of the state to protect or promote community health and the limitations on those powers (21). Topics covered in the health law module were federalism, police power, home rule, public good vs. individual autonomy, the difference between laws and regulations, and the levels and roles of government in the United States. In the second health policy module, the policy development and implementation process was outlined in detail, including the formulation, implementation, modification phases (22). Students were provided examples of how public health policy problems are identified and how these problems get placed on the health policy agenda (i.e., the “window of opportunity”) (23). Also, the basics of how regulators, such as the Centers for Disease Control and Prevention, implement legislation in order to achieve the public health aims within the context of examples. Finally, students consider how to determine the outcomes of existing policy (including the unintended consequences caused by bad policy) lead to policy modification.

Objectives for the advocacy and coalition building module were: (1) define the similarities and differences between advocacy and lobbying for public health issues; (2) explain best practices for effective advocacy including communication and meeting skills with legislators; and (3) describe steps that will build effective coalitions for advocacy efforts. Topics covered in the advocacy module were definitions of advocacy which can be any person or group who advocates, supports, and argues for a cause and lobbying such that a lobbyist is generally a paid representative of a group, organization, or industry who communicates with legislators about specific legislation and expresses the view or opinion of their organization; effective tips for performing successful advocacy including, knowing the difference between regular advocacy and media advocacy such that in media advocacy the target audience are those individuals who can make policy changes; and steps in building a successful coalition. These steps focus on making sure your coalition is broad-based in participation, has a mission, goals and objectives, and stays focused on the advocacy issue during meetings and in-between meetings. Also in the advocacy module students were asked to rank areas of advocacy of which they had the most interest. These areas included: (1) universal motorcycle helmet laws, all ages, all riders; (2) increase funding for human trafficking programs; and (3) ban assault weapons. These public health areas were chosen based on relevance for the geographic area and a long history of difficulties in the passage of related legislation.

For the 3-h online synchronous activity session, 30 students participated through Microsoft Teams videoconferencing application. At the start, a brief review of major components of the modules was delivered by program faculty (1.5 h). Then, students were put into groups based on their interest of topics, as reflected in the results of the modules, for ~60 to 70 min. During this time, the groups of students prepared an advocacy brief to be presented to all IPE participants.

Following the group break-out sessions, the groups reported out to all participants on the following components:

**What is the issue?**

- Why is it important?

**Magnitude of the problem (data)**

- How many people does this impact?
- Have current data to substantiate your position

**Best practices available anywhere**

- Who is doing what about your issue?
- How has it worked?
- How might it affect constituencies?

**Recommendations**

- What are you recommending (beside more money!)?
- Be specific, refer to elements of where this has worked

**Impact/Consequences statement**

- What is the impact of doing nothing?
- What is the main point you need to convey?

**Summary**

- Summarize why your group's issue is important and what you want change-agents to do.

A student representing each group orally presented the group's brief for the course faculty to critique. All student participants also provided feedback to the presenters. Suggestions made by the faculty included the importance of cost information related to interventions, how to reach the broadest audience possible, and connecting with lawmakers through personal accounts or stories.

After the event concluded, an evaluation of the IPE was assessed through an online survey with five largely Likert-type items and one text-based feedback question of the event. The survey questions included:

1. How effective were the Canvas modules in preparing you for the IPE event?
2. The goal of the IPE event was to engage students in a team approach to address a current healthcare issue that could be solved with health policy changes and/or a health advocacy approach. Did this event accomplish its intended goal?
3. Teamwork is one of the four core competencies of interprofessional practice. Did the breakout sessions increase your ability to perform effectively on an interprofessional team?
4. After participating in this IPE event, I am knowledgeable about the foundations of health policy and advocacy.
5. Would you recommend this program to other students in your program?

## Results

Thirty graduate students from the Colleges of Public Health, Nursing, Pharmacy, and Physician Assistant programs participated in the IPE session. Seventeen students were from the College of Public Health (40%), seven students were from the College of Nursing (23%), five students (16.7%) were from the College of Pharmacy, and one student (3.3%) was enrolled in the Physician Assistant Program. Of the 30 participants, 13 responded (43%) to our program evaluation. Of these respondents, four (31%) were from the College of Nursing and nine (69%) were from the College of Public Health. The respondents found the modules very effective (23%) or effective (77%) in preparation for the event. They also agreed (77% responded definitely yes, and 23% responded yes) that the modules met the goal of the session which was to use a team approach to address a current healthcare issue that could be solved with health policy changes and/or health advocacy. For the breakout sessions strengthening teamwork ability, the majority responded affirmatively, with 77% strongly agreeing and 23% somewhat agreeing to this item. Over three-quarters of the respondents (77%) strongly agreed that the event increased their knowledge of policy and advocacy, while 23% somewhat agreed. Finally, all respondents (100%) reported they would recommend the program to other students. In terms of open-ended responses, students remarked that it would be beneficial if they had more time to work on their position paper. This could be accomplished by decreasing the time devoted to the review of concepts at the start of the event since the students were responsible for learning the modules beforehand.

## Discussion

The COVID-19 pandemic has interrupted more traditional IPE educational delivery; however, use of asynchronous modules and a synchronous session proved to be a feasible educational strategy. In concert with the tenants of adult education, this educational experience allowed adult learners to partake in curricula that was problem-centered and for students to be involved with the direction of the course through their teamwork discussions. It also allowed for prior and present occupational and life experiences to be built into the program and discussion, and for the learners to see immediate relevance to their fields. We encourage health programs and disciplines to pursue IPEs with their students and incorporate adult education strategies. Also, IPEs may be helpful for professional nursing, medical, public health, and allied health associations to incorporate into their education efforts.

We plan to develop additional IPEs based on advocacy and policy as a follow-up to this pilot endeavor and will continue to offer these programs as asynchronous modules followed by synchronous activities. We also will continue to evaluate the curricula for efficacy and to focus on increasing the numbers of those individuals who evaluate the program. These methods may include having the evaluation available immediately after the synchronous section or utilizing incentives for completion. While our results may not be generalizable to other interprofessional learning environments, we believe that the foundations of this approach can be translated to various topics in the health professions and tailored for program efforts.

## Data Availability Statement

All datasets presented in this study are included in the article/supplementary material.

## Author Contributions

KL and ZP conceptualized the content of the paper, wrote major sections, and reviewed all versions before submission. SB wrote the IPE history section of the paper and reviewed all revised versions. KL and ZP led the synchronous event and developed the asynchronous modules and activities. SB provided all administrative support. All authors contributed to the article and approved the submitted version.

## Conflict of Interest

The authors declare that the research was conducted in the absence of any commercial or financial relationships that could be construed as a potential conflict of interest.
